# Cs_0.49_NbPS_6_

**DOI:** 10.1107/S1600536810052724

**Published:** 2010-12-24

**Authors:** Eunsil Lee, Yonghee Lee, Hoseop Yun

**Affiliations:** a Division of Energy Systems Research and Department of Chemistry, Ajou University, Suwon 443-749, Republic of Korea

## Abstract

The quaternary thio­phosphate, Cs_0.49_NbPS_6_, caesium hexa­thio­niobiophosphate(V), has been synthesized by the reactive halide flux method. The title compound is isotypic with Rb_0.46_TaPS_6_ and is made up of a bicapped trigonal–biprismatic [Nb_2_S_12_] unit and a tetra­hedral [PS_4_] group. The [Nb_2_S_12_] units linked by the [PS_4_] tetra­hedra form infinite chains, yielding a three-dimensional network with rather large van der Waals gaps along the *c* axis in which the disordered Cs^+^ ions reside. The electrons released by the Cs atoms are transferred to the pairwise niobium metal site and there are substantial inter­metallic Nb—Nb bonding inter­actions. This leads to a significant decrease of the inter­metallic distance in the title compound compared to that in TaPS_6_. The classical charge balance of the title compound may be represented as [Cs^+^]_0.49_[Nb^4.51+^][P^5+^][S^2−^]_4_[S_2_^2−^].

## Related literature

For the synthesis and structural characterization of TaPS_6_, see: Fiechter *et al.* (1980 ▶). For the related quaternary alkali metal thio­phosphates, see: K_0.38_TaPS_6_ and Rb_0.46_TaPS_6_ (Gutzmann *et al.*, 2004*a* ▶) and *A*_2_Nb_2_P_2_S_12_ (*A* = K, Rb, Cs; Gieck *et al.*, 2004 ▶). Quite a few quaternary alkali metal thio­phosphates having similar composition but with different structures have been reported. For compounds with layered structures, see: Gutzmann & Bensch (2003 ▶) for Rb_4_Ta_4_P_4_S_24_; Gutzmann *et al.* (2004*b* ▶) for Cs_4_Ta_4_P_4_S_24_ and Cs_2_Ta_2_P_2_S_12_ and Gutzmann *et al.* (2005) ▶ for K_4_Ta_4_P_4_S_24_. For Rb_2_Ta_2_P_2_S_11_ with a one-dimensional structure, see: Gutzmann & Bensch (2002 ▶).

## Experimental

### 

#### Crystal data

Cs_0.49_NbPS_6_
*M* *_r_* = 380.95
Tetragonal, 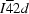

*a* = 15.9477 (3) Å
*c* = 13.2461 (3) Å
*V* = 3368.88 (11) Å^3^
*Z* = 16
Mo *K*α radiation
μ = 5.08 mm^−1^
*T* = 290 K
0.30 × 0.08 × 0.06 mm

#### Data collection

Rigaku R-AXIS RAPID diffractometer
Absorption correction: multi-scan (*ABSCOR*; Higashi, 1995 ▶) *T*_min_ = 0.751, *T*_max_ = 1.000
16125 measured reflections
1929 independent reflections
1843 reflections with *I* > 2σ(*I*)
*R*_int_ = 0.032

#### Refinement

*R*[*F* ^2^ > 2σ(*F* ^2^)] = 0.020
*wR*(*F* ^2^) = 0.047
*S* = 1.06
1929 reflections
81 parameters
Δρ_max_ = 0.84 e Å^−3^
Δρ_min_ = −0.42 e Å^−3^
Absolute structure: Flack (1983 ▶), 851 Friedel pairs
Flack parameter: 0.452 (18)

### 

Data collection: *RAPID-AUTO* (Rigaku, 2006 ▶); cell refinement: *RAPID-AUTO*; data reduction: *RAPID-AUTO*; program(s) used to solve structure: *SHELXS97* (Sheldrick, 2008 ▶); program(s) used to refine structure: *SHELXL97* (Sheldrick, 2008 ▶); molecular graphics: locally modified version of *ORTEP* (Johnson, 1965 ▶); software used to prepare material for publication: *WinGX* (Farrugia, 1999 ▶).

## Supplementary Material

Crystal structure: contains datablocks global, I. DOI: 10.1107/S1600536810052724/si2311sup1.cif

Structure factors: contains datablocks I. DOI: 10.1107/S1600536810052724/si2311Isup2.hkl

Additional supplementary materials:  crystallographic information; 3D view; checkCIF report

## Figures and Tables

**Table:** 

Nb—S1^i^	2.5016 (9)
Nb—S6^ii^	2.5221 (9)
Nb—S1^iii^	2.5238 (8)
Nb—S6^iv^	2.5457 (9)
Nb—S2	2.5457 (9)
Nb—S3	2.5730 (9)
Nb—S5^v^	2.5971 (9)
Nb—S4	2.6266 (9)
Nb—Nb^vi^	3.1236 (5)
P1—S2^i^	2.0300 (11)
P1—S2	2.0300 (11)
P1—S5^vii^	2.0413 (10)
P1—S5^v^	2.0413 (10)
P2—S3^viii^	2.0353 (10)
P2—S3^ix^	2.0353 (10)
P2—S4^ix^	2.0519 (11)
P2—S4^viii^	2.0519 (11)
S1—S1^x^	2.0302 (17)
S6—S6^xi^	2.0264 (17)

**Table:** 

S2^i^—P1—S2	109.11 (7)
S2^i^—P1—S5^vii^	102.92 (3)
S2—P1—S5^vii^	113.21 (4)
S2^i^—P1—S5^v^	113.21 (4)
S2—P1—S5^v^	102.92 (3)
S5^vii^—P1—S5^v^	115.63 (7)

Symmetry codes: (i) 
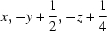
; (ii) 
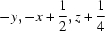
; (iii) 
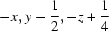
; (iv) 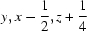; (v) 
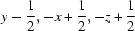
; (vi) 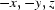; (vii) 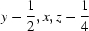; (viii) 
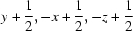
; (ix) 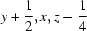; (x) 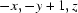; (xi) 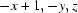.

**Fig. 1.**
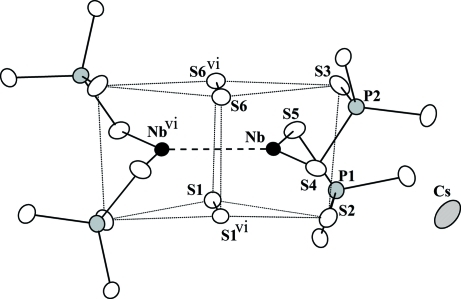
A perspective view of the bicapped trigonal biprismatic [Nb2S12] unit and its neighboring tetrahedral [PS4] groups. The Nb—S bonds have been omitted for clarity, except for the capping S atoms. Displacement ellipsoids are drawn at the 60% probability level. [Symmetry code: (vi) -x, -y, z]

**Fig. 2.**
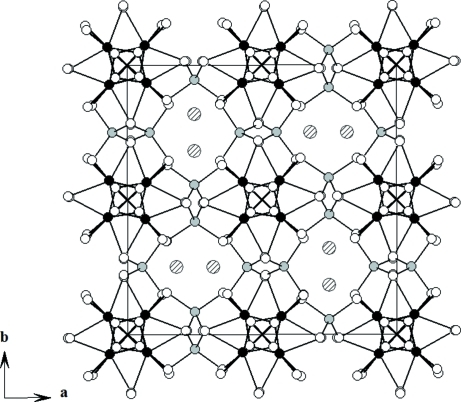
View of Cs0.49NbPS6 along the c axis. Atoms are as marked in Fig. 1.

## supplementary crystallographic information

### Comment

During an effort to find a new phase in the A*_x_*MPS_6_ family (A=alkali metals; *M*=Ta, Nb), a new compound was isolated. Here we report the synthesis and structure of the new quaternary thiophosphates, Cs_0.49_NbPS_6_.

The title compound is isostructural with the previously reported Rb_0.46_TaPS_6_ (Gutzmann *et al.*, 2004*a*). The structure of Cs_0.49_NbPS_6_ is also very similar to that of ANb_2_P_2_S_12_ (A=K, Rb, and Cs, Gieck *et al.*, 2004) prepared from alkali metal sulfide fluxes. The only difference between them lies in the distribution of the alkali metals. There are two crystallographically independent sites for Cs atoms in ANb_2_P_2_S_12_, while we were able to find only one in Cs_0.49_NbPS_6_.

The structure of Cs_0.49_NbPS_6_ is made up of the bicapped trigonal biprismatic [Nb_2_S_12_] unit and the tetrahedral [PS_4_] group. The niobium atom is coordinated by eight sulfur atoms in a distorted bicapped trigonal prismatic arrangement. Two NbS_8_ prisms share a rectangular face to form the [Nb_2_S_12_] dimeric core. Four sulfur atoms sharing rectangular prism faces are in pairs with two disulfide ions, (S—S)^2-^. Each one of the capping sulfur atoms and one of the sulfur atoms at the corner of the [Nb_2_S_12_] unit are bound to a phosphorous atom (Fig. 1). Additional two sulfur atoms from the neighboring [Nb_2_S_12_] units are connected to the phosphorous atoms to complete the [PS_4_] tetrahedral coordination. Each [Nb_2_S_12_] unit connects four phosphorous atoms to build up left- and right-handed helices. These helices interwind to each other forming infinite channels along the [001] direction (Fig. 2). The structural array yields rather large channels, where the Cs^+^ ions reside. The size of the cations is small compared to the diameter of the large channels and the cations can therefore rattle within the channels as indicated by the high anisotropic displacement parameters.

The Nb—S and P—S distances are in good agreement with those found in other related phases (Gutzmann *et al.*, 2004*b*). The interatomic Nb—Nb distance is 3.124 (1) Å which is similar to those in K_0.38_TaPS_6_ (3.142 (2) Å) and Rb_0.46_TaPS_6_ (3.1011 (5) Å). These distances are considerably shorter compared to that in TaPS_6_ (3.361 (1) Å, Fiechter *et al.*, 1980). The electrons released by the Cs atoms are transferred to pair-wise niobium metal sites and there are substantial intermetallic Nb—Nb bonding interactions. Consequently, the classical charge balance of the title compound may be represented as [Cs^+^]_0.49_[*M*^4.51+^][P^5+^][S^2-^]_4_[S_2_^2-^].

### Experimental

Cs_0.49_NbPS_6_ was prepared by the reaction of elements Nb, P, and S by the reactive halide-flux technique. A combination of the pure elements, Nb powder (CERAC 99.999%), P powder (CERAC 99.5%) and S powder (Aldrich 99.999%) were mixed in a fused silica tube in molar ratio of Nb:*P*:*S*=1:1:6 in the presence of CsCl as flux. The mass ratio of the reactants and the alkali halide flux was 1:2. The tube was evacuated to 0.133 Pa, sealed, and heated gradually (60 K/h) to 973 K, where it was kept for 72 h. The tube was cooled to room temperature at the rate 4 K/h. The excess halide was removed with distilled water and shiny black needle-shaped crystals were obtained. The crystals are stable in air and water. Qualitative analysis of the crystals with an EDAX-equipped SEM indicated the presence of Cs, Nb, P, and S. The composition of the compound was determined by single-crystal X-ray diffraction.

### Refinement

(type here to add refinement details)

### Crystal data

**Table:**

Cs_0.49_NbPS_6_	*D*_x_ = 3.004 Mg m^−^^3^
*M**_r_* = 380.95	Mo *K*α radiation, λ = 0.71073 Å
Tetragonal, *I*42*d*	Cell parameters from 14367 reflections
Hall symbol: I -4 2bw	θ = 3.3–27.5°
*a* = 15.9477 (3) Å	µ = 5.08 mm^−^^1^
*c* = 13.2461 (3) Å	*T* = 290 K
*V* = 3368.88 (11) Å^3^	Needle, black
*Z* = 16	0.30 × 0.08 × 0.06 mm
*F*(000) = 2860	

### Data collection

**Table:**

Rigaku R-AXIS RAPID diffractometer	1843 reflections with *I* > 2σ(*I*)
ω scans	*R*_int_ = 0.032
Absorption correction: multi-scan (*ABSCOR*; Higashi, 1995)	θ_max_ = 27.5°, θ_min_ = 3.2°
*T*_min_ = 0.751, *T*_max_ = 1.000	*h* = −20→20
16125 measured reflections	*k* = −20→20
1929 independent reflections	*l* = −16→17

### Refinement

**Table:**

Refinement on *F*^2^	0 restraints
Least-squares matrix: full	*w* = 1/[σ^2^(*F*_o_^2^) + (0.0309*P*)^2^ + 0.1672*P*] where *P* = (*F*_o_^2^ + 2*F*_c_^2^)/3
*R*[*F*^2^ > 2σ(*F*^2^)] = 0.020	(Δ/σ)_max_ = 0.001
*wR*(*F*^2^) = 0.047	Δρ_max_ = 0.84 e Å^−^^3^
*S* = 1.06	Δρ_min_ = −0.42 e Å^−^^3^
1929 reflections	Absolute structure: Flack (1983), 851 Friedel pairs
81 parameters	Flack parameter: 0.452 (18)

### Special details

**Table:**

Geometry. All e.s.d.'s (except the e.s.d. in the dihedral angle between two l.s. planes) are estimated using the full covariance matrix. The cell e.s.d.'s are taken into account individually in the estimation of e.s.d.'s in distances, angles and torsion angles; correlations between e.s.d.'s in cell parameters are only used when they are defined by crystal symmetry. An approximate (isotropic) treatment of cell e.s.d.'s is used for estimating e.s.d.'s involving l.s. planes.

### Fractional atomic coordinates and isotropic or equivalent isotropic displacement parameters (Å^2^)

**Table:**

	*x*	*y*	*z*	*U*_iso_*/*U*_eq_	Occ. (<1)
Cs	0.31826 (2)	0.25	0.125	0.04486 (17)	0.974 (2)
Nb	0.066145 (17)	0.072220 (17)	0.24995 (2)	0.01403 (8)	
P1	0.05549 (7)	0.25	0.125	0.0163 (2)	
P2	0.58291 (7)	0.25	0.125	0.0161 (2)	
S1	0.04769 (5)	0.54216 (5)	0.12737 (6)	0.01871 (18)	
S2	0.12931 (6)	0.14853 (5)	0.09924 (7)	0.02128 (18)	
S3	0.14510 (5)	0.15492 (6)	0.38699 (7)	0.0232 (2)	
S4	0.22010 (5)	0.01370 (5)	0.24947 (7)	0.02042 (18)	
S5	0.28529 (5)	0.48732 (5)	0.25169 (7)	0.02144 (19)	
S6	0.45621 (5)	0.04603 (5)	0.12972 (6)	0.01928 (19)	

### Atomic displacement parameters (Å^2^)

**Table:**

	*U*^11^	*U*^22^	*U*^33^	*U*^12^	*U*^13^	*U*^23^
Cs	0.0218 (2)	0.0690 (3)	0.0438 (2)	0	0	0.0247 (2)
Nb	0.01411 (14)	0.01527 (14)	0.01272 (13)	−0.00014 (9)	−0.00042 (12)	−0.00043 (12)
P1	0.0171 (5)	0.0137 (5)	0.0181 (5)	0	0	0.0005 (5)
P2	0.0191 (5)	0.0140 (5)	0.0153 (5)	0	0	−0.0011 (5)
S1	0.0200 (4)	0.0213 (4)	0.0148 (3)	0.0027 (3)	−0.0025 (4)	−0.0031 (3)
S2	0.0219 (4)	0.0167 (4)	0.0252 (4)	0.0008 (3)	0.0066 (3)	0.0008 (3)
S3	0.0187 (4)	0.0246 (4)	0.0262 (5)	0.0045 (4)	−0.0045 (3)	−0.0115 (4)
S4	0.0200 (4)	0.0236 (4)	0.0177 (4)	0.0059 (3)	−0.0015 (4)	−0.0051 (4)
S5	0.0221 (4)	0.0227 (4)	0.0196 (4)	−0.0066 (3)	0.0028 (4)	−0.0059 (4)
S6	0.0216 (4)	0.0201 (4)	0.0161 (4)	0.0017 (4)	0.0013 (4)	−0.0020 (3)

### Geometric parameters (Å, °)

**Table:**

Cs—S2	3.4374 (10)	P1—S2^i^	2.0300 (11)
Cs—S2^i^	3.4373 (10)	P1—S2	2.0300 (11)
Cs—S3^ii^	3.5468 (9)	P1—S5^xi^	2.0413 (10)
Cs—S3^iii^	3.5468 (9)	P1—S5^ix^	2.0413 (10)
Cs—S4^iv^	3.5646 (9)	P2—S3^iv^	2.0353 (10)
Cs—S4^v^	3.5646 (9)	P2—S3^v^	2.0353 (10)
Cs—S6^i^	3.9274 (9)	P2—S4^v^	2.0519 (11)
Cs—S6	3.9274 (9)	P2—S4^iv^	2.0519 (11)
Cs—S5^i^	4.1733 (8)	S1—S1^xii^	2.0302 (17)
Cs—S5	4.1733 (8)	S1—Nb^i^	2.5016 (9)
Cs—P1	4.1906 (12)	S1—Nb^xiii^	2.5238 (8)
Cs—P2	4.2206 (13)	S3—P2^xiv^	2.0353 (10)
Nb—S1^i^	2.5016 (9)	S3—Cs^xv^	3.5468 (9)
Nb—S6^vi^	2.5221 (9)	S4—P2^xiv^	2.0519 (11)
Nb—S1^vii^	2.5238 (8)	S4—Cs^xiv^	3.5646 (9)
Nb—S6^viii^	2.5457 (9)	S5—P1^xvi^	2.0413 (10)
Nb—S2	2.5457 (9)	S5—Nb^xvi^	2.5971 (9)
Nb—S3	2.5730 (9)	S6—S6^xvii^	2.0264 (17)
Nb—S5^ix^	2.5971 (9)	S6—Nb^xviii^	2.5221 (9)
Nb—S4	2.6266 (9)	S6—Nb^v^	2.5457 (9)
Nb—Nb^x^	3.1236 (5)		
			
S2—Cs—S2^i^	57.52 (3)	S1^i^—Nb—S2	82.33 (3)
S2—Cs—S3^ii^	104.92 (2)	S6^vi^—Nb—S2	154.30 (3)
S2^i^—Cs—S3^ii^	91.80 (2)	S1^vii^—Nb—S2	81.46 (3)
S2—Cs—S3^iii^	91.80 (2)	S6^viii^—Nb—S2	157.94 (3)
S2^i^—Cs—S3^iii^	104.92 (2)	S1^i^—Nb—S3	155.09 (3)
S3^ii^—Cs—S3^iii^	161.04 (3)	S6^vi^—Nb—S3	87.62 (3)
S2—Cs—S4^iv^	151.14 (2)	S1^vii^—Nb—S3	157.05 (3)
S2^i^—Cs—S4^iv^	131.09 (2)	S6^viii^—Nb—S3	87.58 (3)
S3^ii^—Cs—S4^iv^	102.32 (2)	S2—Nb—S3	96.58 (3)
S3^iii^—Cs—S4^iv^	59.903 (19)	S1^i^—Nb—S5^ix^	125.13 (3)
S2—Cs—S4^v^	131.09 (2)	S6^vi^—Nb—S5^ix^	79.61 (3)
S2^i^—Cs—S4^v^	151.14 (2)	S1^vii^—Nb—S5^ix^	79.19 (3)
S3^ii^—Cs—S4^v^	59.903 (19)	S6^viii^—Nb—S5^ix^	125.49 (3)
S3^iii^—Cs—S4^v^	102.32 (2)	S2—Nb—S5^ix^	76.51 (3)
S4^iv^—Cs—S4^v^	58.06 (3)	S3—Nb—S5^ix^	78.13 (3)
S2—Cs—S6^i^	151.57 (2)	S1^i^—Nb—S4	81.33 (3)
S2^i^—Cs—S6^i^	95.896 (19)	S6^vi^—Nb—S4	126.93 (3)
S3^ii^—Cs—S6^i^	62.58 (2)	S1^vii^—Nb—S4	127.11 (3)
S3^iii^—Cs—S6^i^	106.020 (19)	S6^viii^—Nb—S4	82.02 (3)
S4^iv^—Cs—S6^i^	53.620 (18)	S2—Nb—S4	78.35 (3)
S4^v^—Cs—S6^i^	67.27 (2)	S3—Nb—S4	74.12 (3)
S2—Cs—S6	95.896 (19)	S5^ix^—Nb—S4	139.76 (3)
S2^i^—Cs—S6	151.57 (2)	S1^i^—Nb—Nb^x^	51.888 (19)
S3^ii^—Cs—S6	106.020 (19)	S6^vi^—Nb—Nb^x^	52.29 (2)
S3^iii^—Cs—S6	62.58 (2)	S1^vii^—Nb—Nb^x^	51.25 (2)
S4^iv^—Cs—S6	67.27 (2)	S6^viii^—Nb—Nb^x^	51.61 (2)
S4^v^—Cs—S6	53.620 (18)	S2—Nb—Nb^x^	128.30 (2)
S6^i^—Cs—S6	111.87 (3)	S3—Nb—Nb^x^	135.12 (2)
S2—Cs—S5^i^	54.738 (19)	S5^ix^—Nb—Nb^x^	108.56 (2)
S2^i^—Cs—S5^i^	110.88 (2)	S4—Nb—Nb^x^	111.67 (2)
S3^ii^—Cs—S5^i^	92.93 (2)	S2^i^—P1—S2	109.11 (7)
S3^iii^—Cs—S5^i^	89.45 (2)	S2^i^—P1—S5^xi^	102.92 (3)
S4^iv^—Cs—S5^i^	114.78 (2)	S2—P1—S5^xi^	113.21 (4)
S4^v^—Cs—S5^i^	78.539 (18)	S2^i^—P1—S5^ix^	113.21 (4)
S6^i^—Cs—S5^i^	144.617 (19)	S2—P1—S5^ix^	102.92 (3)
S6—Cs—S5^i^	47.615 (17)	S5^xi^—P1—S5^ix^	115.63 (7)
S2—Cs—S5	110.88 (2)	S2^i^—P1—Cs	54.56 (4)
S2^i^—Cs—S5	54.738 (19)	S2—P1—Cs	54.56 (4)
S3^ii^—Cs—S5	89.45 (2)	S5^xi^—P1—Cs	122.18 (4)
S3^iii^—Cs—S5	92.93 (2)	S5^ix^—P1—Cs	122.18 (4)
S4^iv^—Cs—S5	78.539 (18)	S3^iv^—P2—S3^v^	111.30 (8)
S4^v^—Cs—S5	114.78 (2)	S3^iv^—P2—S4^v^	115.55 (4)
S6^i^—Cs—S5	47.615 (17)	S3^v^—P2—S4^v^	100.12 (3)
S6—Cs—S5	144.616 (19)	S3^iv^—P2—S4^iv^	100.12 (3)
S5^i^—Cs—S5	165.52 (3)	S3^v^—P2—S4^iv^	115.55 (4)
S2—Cs—P1	28.759 (15)	S4^v^—P2—S4^iv^	114.91 (8)
S2^i^—Cs—P1	28.759 (15)	S3^iv^—P2—Cs	124.35 (4)
S3^ii^—Cs—P1	99.482 (14)	S3^v^—P2—Cs	124.35 (4)
S3^iii^—Cs—P1	99.482 (14)	S4^v^—P2—Cs	57.46 (4)
S4^iv^—Cs—P1	150.970 (14)	S4^iv^—P2—Cs	57.46 (4)
S4^v^—Cs—P1	150.970 (14)	S1^xii^—S1—Nb^i^	66.74 (3)
S6^i^—Cs—P1	124.066 (13)	S1^xii^—S1—Nb^xiii^	65.60 (3)
S6—Cs—P1	124.066 (13)	Nb^i^—S1—Nb^xiii^	76.86 (3)
S5^i^—Cs—P1	82.760 (13)	P1—S2—Nb	91.14 (3)
S5—Cs—P1	82.760 (13)	P1—S2—Cs	96.69 (4)
S2—Cs—P2	151.241 (15)	Nb—S2—Cs	119.61 (3)
S2^i^—Cs—P2	151.241 (15)	P2^xiv^—S3—Nb	93.32 (4)
S3^ii^—Cs—P2	80.518 (14)	P2^xiv^—S3—Cs^xv^	100.09 (3)
S3^iii^—Cs—P2	80.518 (14)	Nb—S3—Cs^xv^	157.95 (4)
S4^iv^—Cs—P2	29.030 (14)	P2^xiv^—S4—Nb	91.38 (3)
S4^v^—Cs—P2	29.030 (14)	P2^xiv^—S4—Cs^xiv^	93.51 (4)
S6^i^—Cs—P2	55.934 (13)	Nb—S4—Cs^xiv^	115.76 (3)
S6—Cs—P2	55.934 (13)	P1^xvi^—S5—Nb^xvi^	89.43 (3)
S5^i^—Cs—P2	97.240 (13)	P1^xvi^—S5—Cs	147.09 (5)
S5—Cs—P2	97.240 (13)	Nb^xvi^—S5—Cs	108.99 (3)
P1—Cs—P2	180	S6^xvii^—S6—Nb^xviii^	67.04 (3)
S1^i^—Nb—S6^vi^	104.18 (3)	S6^xvii^—S6—Nb^v^	65.82 (3)
S1^i^—Nb—S1^vii^	47.65 (4)	Nb^xviii^—S6—Nb^v^	76.10 (3)
S6^vi^—Nb—S1^vii^	84.90 (3)	S6^xvii^—S6—Cs	170.46 (6)
S1^i^—Nb—S6^viii^	84.86 (3)	Nb^xviii^—S6—Cs	118.42 (3)
S6^vi^—Nb—S6^viii^	47.14 (4)	Nb^v^—S6—Cs	106.98 (3)
S1^vii^—Nb—S6^viii^	102.86 (3)		

Symmetry codes: (i) *x*, −*y*+1/2, −*z*+1/4; (ii) −*x*+1/2, −*y*+1/2, *z*−1/2; (iii) −*x*+1/2, *y*, −*z*+3/4; (iv) *y*+1/2, −*x*+1/2, −*z*+1/2; (v) *y*+1/2, *x*, *z*−1/4; (vi) −*y*, −*x*+1/2, *z*+1/4; (vii) −*x*, *y*−1/2, −*z*+1/4; (viii) *y*, *x*−1/2, *z*+1/4; (ix) *y*−1/2, −*x*+1/2, −*z*+1/2; (x) −*x*, −*y*, *z*; (xi) *y*−1/2, *x*, *z*−1/4; (xii) −*x*, −*y*+1, *z*; (xiii) −*x*, *y*+1/2, −*z*+1/4; (xiv) −*y*+1/2, *x*−1/2, −*z*+1/2; (xv) −*x*+1/2, −*y*+1/2, *z*+1/2; (xvi) −*y*+1/2, *x*+1/2, −*z*+1/2; (xvii) −*x*+1, −*y*, *z*; (xviii) −*y*+1/2, −*x*, *z*−1/4.
